# Cases of Empyema Cured by Operation

**Published:** 1833-09

**Authors:** 


### EMPYEMA.

### *Cases of Empyema cured by Operation.*
### [*Gazette Médicale de Paris.*]

*Empyema, following Variola, cured by Operation.*

Case I. Pasquet Benjamin, twenty-five years of age, of a weak constitution, was admitted into the hospital at Tours, on the 1st January, with intense fever and variolous eruption. So great was the agitation under which he laboured, that he could not be kept in bed. The eruption was imperfect. At the end of January the following symptoms existed: short, difficult, and rapid breathing; a sensation of oppression and heaviness at the diaphragm; a sense of suffocation upon the least motion; constant cough; incapability of lying on the right side. The left side of the chest was sensibly distended; the intercostal spaces being much broader than natural, and projecting at the lower and posterior parts. The heart was thrown towards the right side, and its pulsations were perceptible at the inferior part of the thorax. When the patient moved, fluctuation and undulation were evident even to the sight and hearing, without having recourse to auscultation.

February 1st. The operation for empyema was performed by M. Herpin. The quantity of pus thrown out had so greatly depressed the diaphragm, that the incision was made between the tenth and eleventh ribs, at about an inch from the costal angle, where the fluctuation was most evident. An incision was made in a fold of the skin, about two inches long, and parallel to the direction of the intercostal space. The pleura being exposed, an opening of two or three lines long was made into it with a bistoury. Liquid, inodorous pus, mingled with serous shreds, at first escaped by jets, and with force. When about a pint was evacuated, the wound was dressed with a compress of lint, with holes in it to admit of still further discharge. For several days the bed of the patient was soaked with pus. The discharge became more and more fœtid, and blackened the catheter which was introduced each time the wound was dressed. The edges of the wound were tumefied and inflamed, and the patient was in pain and much agitated.

His face became pale and anxious, eyes sunken, skin of a dull colour, and there was great general depression.

In this state he continued until the middle of March; he was then directed to take kino and quinine in infusion, and an injection was thrown into the wound of decoction of kino, with a few drops of solution of chlorate of lime. From this time he gradually improved; the pus became whiter, thicker, and of good quality. In a few days there was little or no suppuration, and on the 2d of May the injections were discontinued.

Three days afterwards, the patient, having eaten more than usual, was attacked with shivering and fever, and spasms of the abdominal muscles, particularly on the left side. The face and feet were œdematous, and the oppression and pain of the side returned as violently as at first.

May 6th. A large quantity of bloody pus was evacuated by means of a catheter, which was now again discoloured. The injections were repeated; low diet. He again improved. The pus now seemed to concentrate itself at the bottom of the pleural cavity, and grumous and fibrinous clots still passed through the catheter.

Towards the end of May there was very little discharge, and now, in order to introduce the catheter to the bottom of the purulent cavity, (about four or five inches deep from the wound,) it was necessary to overcome a resistance, which appeared to depend upon adhesion of the lungs with the pleura of the ribs and diaphragm.

May 30th. The injections were discontinued; the kino mixture was again given; and a blister was applied to the nape of the neck, and kept open for some days. From this period the patient was free from fever or oppression, and he gradually grew fat, and his skin assumed a healthier colour.

On the 2d of July he was presented to the Medical Society of Tours. There was perfect resonance on the right side of the chest; the left side also gave a clear sound at the upper part, but at the inferior part, and around the spot where the operation had been performed, the sound was dull. It is to be observed, that this patient never expectorated pus, although he had coughed a good deal, and his breathing was impeded.

Case II. *Empyema after Measles: Operation; Cure.*

This was the case of a child seven years of age, who had had measles, and been much neglected some days after the appearance of the eruption. Catarrh followed, and subsequently empyema, which caused a projection of the thorax near the inferior angle of the left scapula. M. Herpin made an opening with potassa fusa, and afterwards threw in injections with barley-water and honey. The suppuration continued for six weeks; the wound then closed, and soon after the little patient complained of oppression and sense of suffocation. The wound was again opened, and suppuration went on for several weeks. The parents confessed that they had formerly laboured under syphilis. M. H. prescribed the following medicine: Syrup of sarsaparilla, with deuto-chlorate of mercury, in appropriate doses. In a short time the suppuration diminished, the wound cicatrized, and the child was restored to health. The side of the thorax which had been affected did not, however, increase in size as in the other, and this young person, who is now (1832) twenty-three years old, is a little deformed in shape, although in the enjoyment of good health.

In both these cases the empyema arose from the same cause—repercussion of an exanthematous disease. They were both treated by making an opening which permitted the air to enter freely into the chest, and both did well. Here the resemblance between the two stops. It did not appear that the child experienced such severe symptoms as the man; but the latter had a relapse, and was only saved by the treatment described. M. Herpin makes but few remarks upon these cases: he merely states, that he prescribed mercurials because he has always found them efficacious in removing the bad effects of the morbillous virus.

Case III. *Empyema after several Attacks of Peripneumonia: Operation; Cure.*

A soldier, æt. twenty-six, after several attacks of peripneumonia, was affected with empyema, which caused a projection below the inferior angle of the right scapula. He entered the hospital the 27th March, and on the 23d of April an opening was made into the chest. An ichorous discharge of pus followed, which afterwards became so fetid that the patients all left the ward when he was dressed. Fever succeeded, which placed the patient in danger for several days. He took internally decoction of bark; and an injection of the same mixture was used, with a small quantity of a solution of chlorate of lime. The whole surface of the body was afterwards covered with a miliary eruption; the suppuration diminished, and was less fetid; the eruption died away; the fever subsided. He became convalescent, and left the hospital perfectly cured on the 22d of May.

To these three cases, for which we are indebted to M. Herwin, we add the following, from the *Gazette Scientifique de Seine-et-Oise;* which is still more remarkable, on account of its various complications, and the length of time the discharge continued.

Case IV. *Symptoms of Constitutional Syphilis; Caries of the Ribs; Acute Pleuro-pneumonia; Empyema. Operation, followed by a Discharge for two years; Cure.*

M. R., a cavalry officer, æt. twenty-eight, of lymphatic constitution, had frequently had syphilis. He was admitted into the

Hospice de Versailles on the 19th August. He was treated for abscesses in different parts of the body, and went out convalescent November 6th. On the 22d he was again admitted, on account of a tumor situated on the left side of the chest, above the sixth rib, which had appeared without any assignable cause. It was red, soft, and painful. Forty leeches were applied in two days, but the pain was not relieved. On the third day an incision was made, and it was ascertained, upon the introduction of a sound, that there was caries of the rib. The pain ceased for a very short time, but returned on the following day. There were now dyspnœa, dry and frequent cough, striated expectoration, and in fact every symptom of intense pleuro-pneumonia. He was bled, and the next day was much exhausted and insensible. Revulsive remedies were employed. The right side of the chest and the neck were attacked with erysipelas. Delirium, and increased difficulty of breathing.

Notwithstanding this complication of severe symptoms, the pulmonary affection was soon relieved, and the expectoration was easier. The respiratory sound was heard in the right lung, but in the left it was feared that there was either congestion or effusion; and the subsequent dilatation of this side of the chest removed all doubt upon this point.

As soon as the inflammatory symptoms had entirely subsided, a caustic issue was made. When the slough separated, above two pints of yellowish serum were discharged, in which was floating concreted albumen, which, by blocking up the opening, prevented, at moments, the exit of the fluid. A plug was afterwards inserted into the wound, which was covered with linen with holes made in it. In the course of the next few hours about ten ounces of fluid were evacuated; but, although the patient was much relieved, the suppuration continued fetid and abundant: even the expectoration was purulent. Injections of solution of chlorate of lime were employed, and immediately after the patient complained of tasting the chlorate in the throat. Reduced as he was to a state of emaciation, he was now attacked with acute articular rheumatism, which rendered it necessary to bleed him several times. The pains in the joints were removed, but inflammation of the periosteum remained upon the fibula and tibia. The former went off; but the latter terminated in an abscess, and left the bone exposed to the extent of four or five inches. In six weeks this wound was healed. The two abscesses in the thorax, namely, that of the carious rib, and that from the empyema, lasted a much longer time.* Nearly

* When the ribs are carious in cases of empyema, the cure is always difficult and tedious. A modern writer indeed informs us that, when the inside of the rib is extensively carious, or when the caries is near the junction of the bone to the spine, the fistula is incurable. (Lassus, Pathologie Chirurgicale, t. i. p. 128, edit. 1809.) Another surgeon of great experience recommends us to endeavour to separate the diseased bone, either by cutting it away or by the trephine. (Pelletan, Clinique Chir., t. iii. p. 253.)—Editors.

two years elapsed before the discharge from the latter ceased, but ultimately the patient left the hospital perfectly cured.

It did not appear that the venereal taint of this patient rendered mercurials necessary. He recovered, notwithstanding every circumstance appeared unfavourable: his temperament, his previous diseases, and the various complications of his case. The appearance of the fluid discharged was also bad; for it has been remarked, that there is a greater chance of effecting a cure in empyema when the fluid is purulent than when it is serous.* Both the skilful surgeons who have recorded these cases employed the caustic potash to give exit to the fluid. At no very distant period, abscesses of the pleura were considered as totally distinct from ordinary abscesses. The principal object was to prevent the admission of air; and, although the old opinions upon the subject are now a good deal modified, the use of the caustic issue in such cases has not been before mentioned. We perceive, however, that this mode of making an opening is not more dangerous than an incision by a bistoury.

---

### CRITICAL ANALYSES.

* Sir ASTLEY COOPER states that he has only known one operation for hydrothorax terminate successfully. This he considers by no means surprising, as the collection of fluid is the result of disease of the thoracic viscera, the heart, or lungs, &c. (Lectures, vol. ii. p. 385.)—EDITORS.

